# Nano-cellulose derived bioplastic biomaterial data for vehicle bio-bumper from banana peel waste biomass

**DOI:** 10.1016/j.dib.2016.05.029

**Published:** 2016-05-25

**Authors:** A.B.M. Sharif Hossain, Nasir A. Ibrahim, Mohammed Saad AlEissa

**Affiliations:** a Program of Biotechnology, Department of Biology, Faculty of Science, University of Hail, Saudi Arabia; b Department of Biology, Faculty of Science, AI-Imam Muhammad Ibn Saud Islamic University, Saudi Arabia

**Keywords:** Nano-celluloses, Biopolymer, Banana peel waste, Biobumper

## Abstract

The innovative study was carried out to produce nano-cellulose based bioplastic biomaterials for vehicle use coming after bioprocess technology. The data show that nano-cellulose particle size was 20 nm and negligible water absorption was 0.03% in the bioplastic. Moreover, burning test, size and shape characterizations, spray coating dye, energy test and firmness of bioplastic have been explored and compared with the standardization of synthetic vehicle plastic bumper following the American Society for Testing and Materials (ASTM). Tensile test was observed 120 MPa/kg m^3^. In addition to that pH and cellulose content were found positive in the bioplastic compared to the synthetic plastic. Chemical tests like K, CO_3_, Cl_2,_ Na were determined and shown positive results compared to the synthetic plastic using the EN-14214 (European Norm) standardization.

## Specialization Table

**Table:** 

Subject area	Biological Chemistry
More specific subject area	Bioplastic biomaterial from banana peel biomass
Type of data	Table and figure
How data was acquired	SEM, pH meter, Tensile test was performed by Universal Test Machine, absorption test, burning test, crack test, energy test, chemical test by ASTM and EN standard
Data format	Raw data collection and analysis
Experimental factors	Samples pyrolysis, acid hydrolysis
Experimental features	3 Replicates were used in the experiment as Complete Randomized Design (CRD). The sample was selected randomly from the different lots
Data source location	Hail city, Saudi Arabia
Data accessibility	Data are presented in this article

## Value of the data

• The data are an innovative and important in the research area of nano-cellulose based bio-plastic biomaterials utilizing banana peel waste biomass in the pharmaceuticals, medical, biomedical and bioengineering aspect.
• Innovative information regarding biopolymer production from biomass has been explored. The data can be most useful for the researcher, research student and academician to acquire innovative knowledge.
• Identification of the suitability of produced nano-cellulosed based bio-plastic materials plays a significant role for the researcher in further studies for automotive biobumper production.

## 1 Data

**Fig. 1.1:**
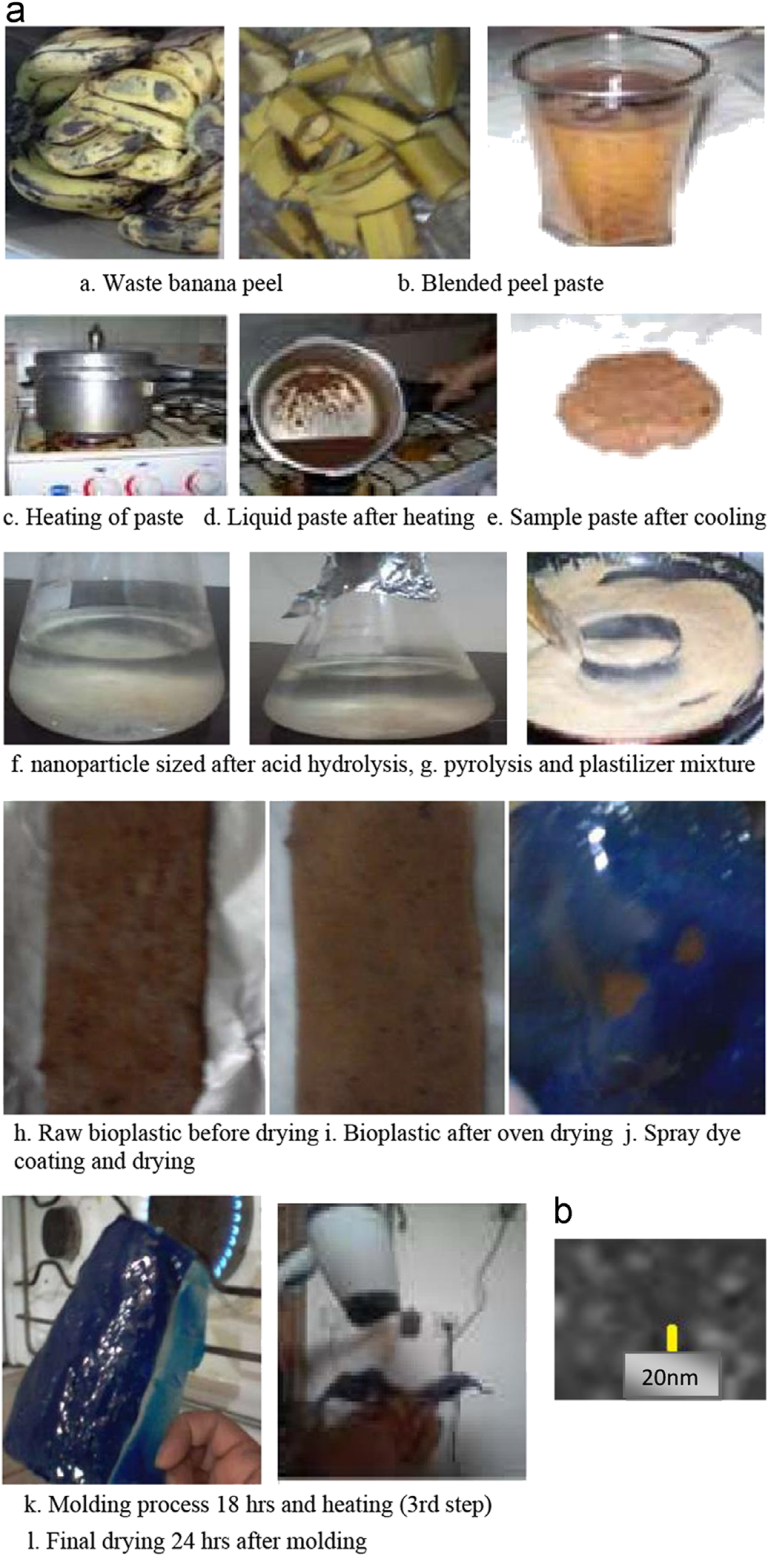
(a) Procedure of nano-bioplastic and biobumper preparation. (b) Nanocellulose.

**Fig. 1.2:**
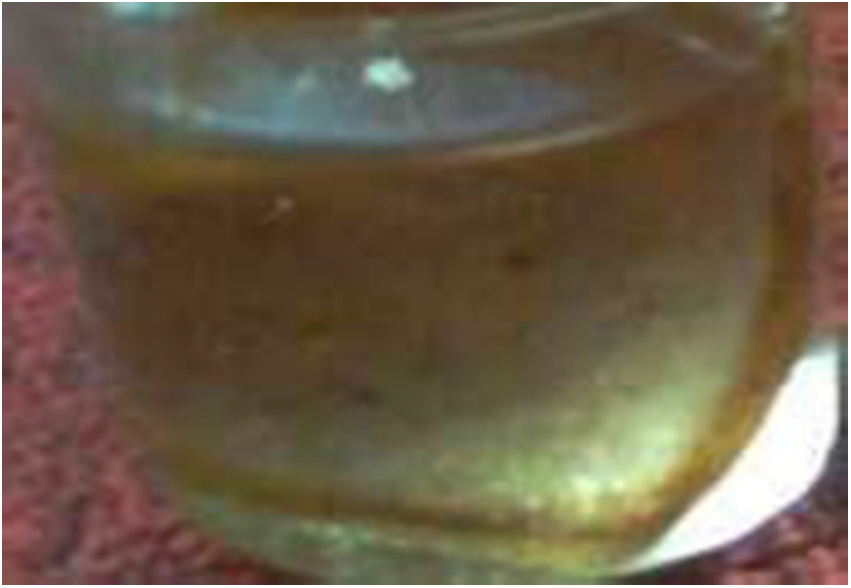
Absorption test.

**Fig. 1.3:**
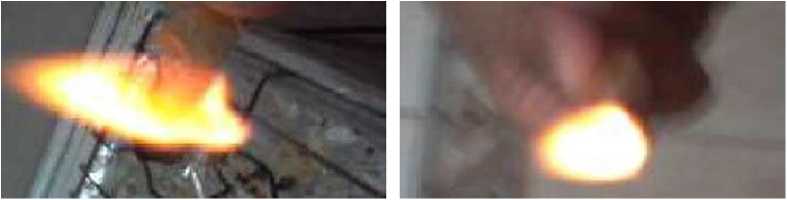
Burning test image.

**Fig. 1.4:**
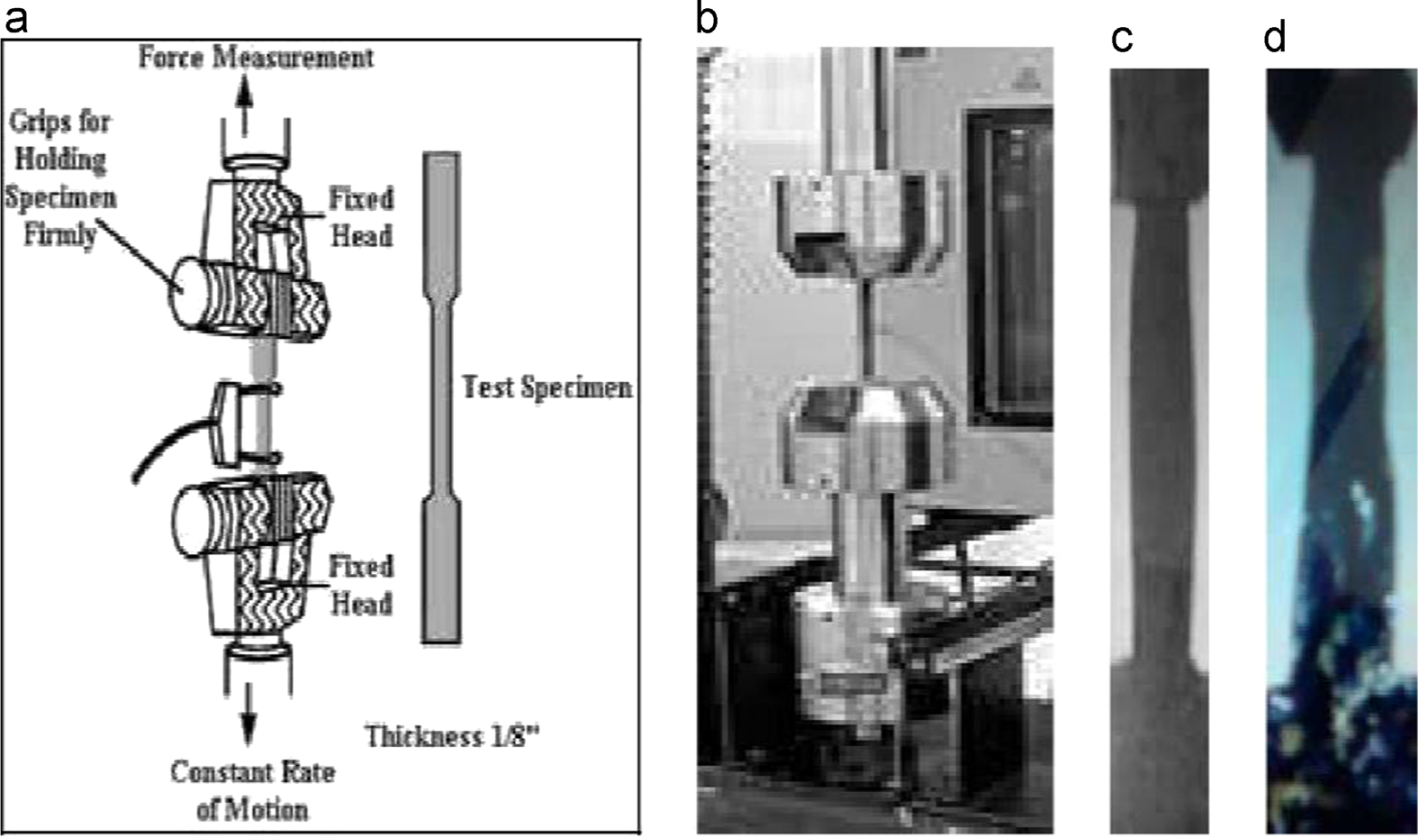
(a) Universal Test Machine procedure, (b) Universal Test Machine, (c) plastic bumper, and (d) bioplastic.

**Fig. 1.5:**
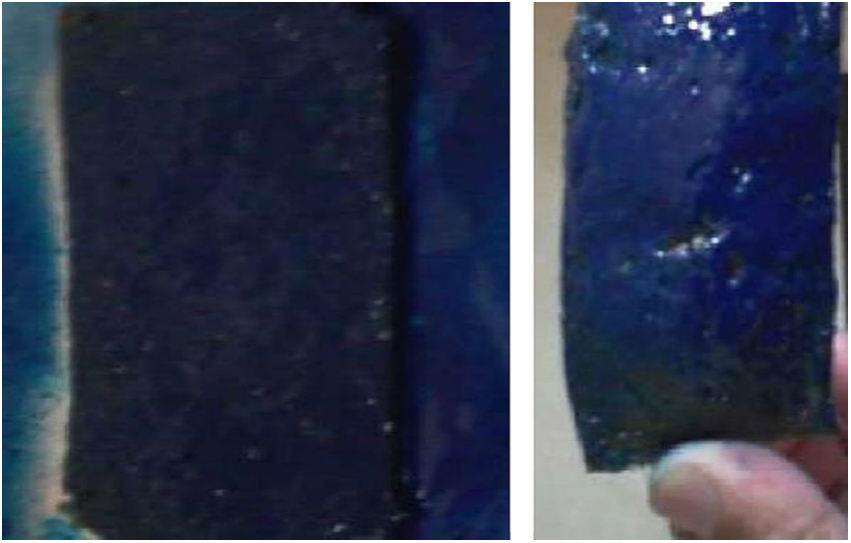
Nano-bioplastic with spray coating test.

The data have been shown the sample collection, preparation and biochemical process in Fig. 1.1, Fig. 1.2, Fig. 1.3, Fig. 1.4, Fig. 1.5. Fig. 1.1a shows the procedure of nano-bioplastic and biobumper preparation. Moreover, Fig. 1.2 demonstrates the absorption test of nano-cellulose based bioplastic. Fig. 1.3 indicates the burning test image. The Universal Test Machine procedure is denoted in Fig. 1.4. Besides, nano-bioplastic having spray coating test has been exhibited (Fig. 1.5). From the data it had been seen that nano-cellulose particle size was 20 nm found in the bioplastic. It was considered for plastic bumper as mentioning the result of ASTM (0–0.16) (Table 1.1). In addition to that burning test (Table 1.2), spray coating dye (Table 1.3), size and shape characterizations (Table 1.4), energy test (Table 1.5), firmness test (Table 1.6) were found positive and standard in bioplastic compared to the synthetic plastic following ASTM standardization. Tensile test was observed 120 MPa/kg m^3^ (Table 1.7). In addition to that chemical analysis like K^+^, CO_3_^−^, Cl_2_, Na were determined (Table 1.8) and shown positive results compared to the synthetic plastic in the laboratory using the EN (166) standardization (Table 1.9).

## 2 Experimental design, materials and methods

### 2.1 Sample collection and preparation

A total of 10 kg waste banana samples (*Musa acuminata)* were collected from the local market, Hail city, KSA. Afterwards, banana peel was removed and washed to ensure proper cleaning. Washed peel was sliced with scissors, then the sliced peel was blended using conventional blender. After blending, the sample was ground again with mortar and pestle, to get a fine mixture and put it into the beaker.

### 2.2 pH determination

The pH was determined using Horiba Scientific pH meter.

### 2.3 Cellulose determination

#### 2.3.1 Dinitrosalicylic acid (DNS) method for cellulose determination

Cellulose content was determined by utilizing 3,5-dinitrosalicylic acid (DNS). A standard curve was drawn by measuring the absorbance of known concentration of cellulose solution at 575 nm. DNS reagent consisted of 1% dinitrosalicylic acid, 0.2% phenol, 0.05% sodium sulfite and 1% sodium hydroxide. To measure cellulose content, 3 ml of unknown cellulose solution was poured into a test tube followed by addition of 3 ml of DNS reagent. The test tubes were then heated in boiling water bath for 15 min. 1 ml of 40% potassium sodium tartrate solution was then added prior to cooling the sample. All test tubes were then cooled under running tap water and its absorbance was measured at 575 nm.

### 2.4 Samples pyrolysis

Blended and ground sample was heated at 150 °C in pressure cooker for 2 h at 30 psi until the sample became liquid paste. After heating, the liquid paste sample was cooled down.

### 2.5 Acid hydrolysis

Paste sample was hydrolyzed (100 ml/50 g sample) by hydrochloric acid (HCl, 99%) to make it micro to nano size particle for 8 h. The water bath was used during the hydrolysis came after in bioprocess technique. After 8 h, the samples were separated using separation funnel and washed by distilled water.

### 2.6 Nanoparticle measurement

Nano particle size of the sample was measured by employing Scanning Electron Microscopy (SEM).

### 2.7 Plastilizer and mixture of chemicals

Chlorinated paraffin liquid plasticizers (1:8 of sample), 5% acetic acid (5 ml/100 g sample), 5 ml/100 g (polyvinyl chloride), cellulose (25%) and 25% starch powder, 5% film forming agent (toluene, Phthalates ester) and 10% water were added with the 1000 g of samples. Later 10 ml/100 g polyvinyl chloride (PVC) and glycerin were added with the mixture of samples and kept for 10 min to mix up well. The dye (benozophenone and stearalkonium) was mixed and kept for 5 min to mix up well and then the mixture was heated implementing pyrolysis method following ASTM standard [1] (at 150 °C in the oven for 30 min; at 30 psi pressure) until visual plasticity in the oven for bioplastic material. The samples were taken out from the oven and stored in room temperature (28 °C) to cool down for 10 min. Afterwards, it was put in the exposed aluminum foils for few hours to make it dry bioplastic. Then it was set for molding process. Finally it was oven dried at 80 °C. The bioplastic was used for different test for fitness.

### 2.8 Molding in the laboratory

The bio-plastic sample was molded using manually prepared mold former and burner for 24–30 h drying and shaping of vehicle bumper. Dryer was used every 5 h to form a curve shape of the bio-bumper. It was kept at −5 °C for one day and taken out from the deep freezer, dried by dryer and kept for hardening at normal temperature.

### 2.9 Fitness testing of bioplastic

#### 2.9.1 Absorption test as ASTM D570 [2]

For the water absorption test, the specimens were dried in an oven for a specified time and temperature and then placed into the desiccator to cool. Immediately upon cooling the specimens were weighed. The material was then merged in water upon conditions, often 23 °C for 24 h. Specimens were removed, patted dry with a lint free cloth, and weighed (Fig. 1.2). Diameter of disk was 5 cm and 2 mm thick. Water absorption was calculated (Table 1.1).

#### 2.9.2 Burning test

Bioplastic was burnt by using gas burner. Odor, color of flame, speed of burning and spark were observed by visual observation and compared with the synthetic bumper by ASTM D3801 [3] (Fig. 1.3).

#### 2.9.3 Tensile/tension test

Tensile test was performed by Universal Test Machine for bioplastics as ASTM D5083 [4].

##### 2.9.3.1 Test procedure

Specimens were placed in the grips of a Universal Test Machine at a specified grip separation and pulled until failure. For ASTM D5083 [4], the test speed was determined by the material specification. The default test speed was 5 mm/min (0.2 in./min), but modulus determinations were made at 2 mm/min (0.079 in./min). An extensometer or strain gauge was used to determine elongation and tensile modulus. Depending upon the reinforcement and type, testing in more than one orientation might be necessary. Max load capacity was 50 kN/m^2^.

##### 2.9.3.2 Sample size

The standard specimen for ASTM D5083 had a constant rectangular cross section, 25 mm (1 in.) wide and at least 250 mm (10 in.) long. Thickness was between 2 mm (0.079 in.) and 14 mm (0.55 in.). Optional tabs could be bonded to the ends of the specimen to prevent gripping damage. Inter-tek PTL could machine the specimens from larger samples and bond tabs if required (Fig. 1.4). Tensile strength (MPa or psi) was displayed from tensile test.

#### 2.9.4 Shape and size test

The specimen was continuously beaten by hammer for 2 min and pulled on for 5 min. There was no change of its shape and size (Fig. 1.5).

#### 2.9.5 Energy test

Energy was tested for the bioplastic based bumper using the following equation:

The energy=1/2*mv*^2^; *m*=mass of hammer, *V* (velocity)=m/s, energy=? J (Table 1.5).

#### 2.9.6 Firmness test (bore test)

Bioplastic was hit with a 2 kg hammer on the screw set on the bioplastic. Hammering was completed after 5 min.

#### 2.9.7 Crack test

A 10 kg weight (5 J of energy) was dropped on to the bioplastic from 1 m height. There was no crack on it. (5 J of energy was calculated by the following equation: energy=1/2*mv*^2^; *m*=mass of stone=10 kg, *V* (velocity)=*m*/*s*=1/1, *m*=distance meter, *s*=second. Therefore, energy=1/2×10×1=5 J.)

#### 2.9.8 Spray coating test

Spray coating dye was used as the mode of application. It was attached properly with plastic and dried after 1 h.

#### 2.9.9 Chemical test

Chemical test like K, CO_3_, Cl_2,_ Na were determined by using different meters and exhibited positive results (Table 1.8) compared to the synthetic plastic in the laboratory using the EN (14214) [5] standardization.

#### 2.9.10 Statistical analysis

Standard deviation was calculated and standard error was analyzed using 3 replicates of the samples where necessary (*n*=3).

## Figures and Tables

**Table 1.1:** Determination of water absorption by ASTM D570.

Materials	Water absorption	ASTM D570
		Water absorption
Bioplastic bumper	0.03%	Water absorption by
Plastic bumper	0–0.16%	ASTM is 0-0.16%.

**Table 1.2:** Burning test according to the ASTM D3801.

Materials	Odor	Color of flame	Speed of burning	Spark or not
Bioplastic bumper	Low odor	Yellow–orange	Slow	Spark
Plastic bumper (by ASTM D3801)	Low odor	Yellow–orange	Slow	Spark

**Table 1.3:** Spray coating dye was used as the mode of application by ASTM B 117

Materials	Spray coating test	ASTM B117
	(Drying time)	Maimum 2 hours
Bioplastic bumper	1.5 h	
Plastic bumper	2 h (max).	
By ASTM B 117		

**Table 1.4:** Determination of size and shape characteristics

Materials	Size and shape	ASTM A500
Bioplastic bumper	No swell or shrink	Resistant characters
Plastic bumper		
By ASTM A 500	No swell or shrink	

**Table 1.5:** Energy test of bioplastic that can be used for bumper

Materials	Energy	ASTM E1886
	E = 1/2x MxV2	
Bioplastic bumper	2.5 Joule	
Plastic bumper		
By ASTM E 1886	1.75-25 Joule	

**Table 1.6:** Firmness test represented by bore and crack test.

	Bore and crack test	
Materials	Firmness test	Firmness test
	Bore test ASTM D2925	Crack test by ASTM D5419
Bioplastic bumper	No bore symptom	No crack symptom
Plastic bumper	No bore symptom	No Crack symptom

**Table 1.7:** Determination of tensile test by using ASTM by ASTM D5083

Materials	Tensile strength	Tensile Modulus (GPa)
	(MPa/kg.m3)	
Bioplastic bumper	120.0	1.1
Plastic bumper		
By ASTM D5083	70-230 (ASTM)	1.0- 3.0 (ASTM)

**Table 1.8:** pH and Cellulose determination.

		pH and Cellulose determination	
Test		pH	Cellulose
Banana samples		7.7±0.01	39.4%±0.1
Plastic	sample	Alkaline ≥ 7	(It is zero if from gas or
			oil sample, if from
			cellulose sample it is 20-
			40%)

Mean ±SE (standard Error, n=3)

**Table 1.9:** Chemical element test.

Chemical element	Banana sample based bioplastic (PPM)	Plastic by EN (European Standard EN166)
K	10±0.5	10
Na	5.1±0.2	5
Cl_2_	0.67±0.01	2
CO_3_	132±2.0	5–440

Mean±standard error (SE, *n*=3).
