# Supplementary material for: Nano-cellulose derived bioplastic biomaterial data for vehicle bio-bumper from banana peel waste biomass
Source: Data Brief. 2016 May 25;8:286–94. doi: 10.1016/j.dib.2016.05.029 (PMC4906129; doi:10.1016/j.dib.2016.05.029)
Supplement: Supplementary file 1 — Supplementary material [file mmc1.docx]

Conflict of Interest Form

Dear Editor

I would like to inform you that we do not have any conflict of interest in publishing the data in Brief.

Regards

Dr. ABM Sharif Hossain

(On behalf of all authors)
